# Analysis of *Acanthopanax giraldii* Harms Polysaccharide II Composition and Its Immune-Protective Role in a Cyclophosphamide-Induced Immunosuppressive Mice Model

**DOI:** 10.1155/2021/3387396

**Published:** 2021-07-31

**Authors:** Xiaoxiao Guo, Huihui Hao, Aixia Meng, Cai Xu, Yuan Ma, Fengxiang Sun, Yong Chen

**Affiliations:** ^1^School of Basic Medicine, Weifang Medical University, Weifang 261042, China; ^2^Affiliated Hospital of Weifang Medical University, Weifang 261042, China; ^3^School of Bioscience and Technology, Weifang Medical University, Weifang 261042, China

## Abstract

*Acanthopanax giraldii* Harms is commonly used in traditional Chinese medicine to treat rheumatism, improve joints, and strengthen muscles and bones. The polysaccharides present in *A. giraldii* Harms contain major bioactive substances, which have antioxidant, anticancer, and antiviral activities. In this study, the structural characterization of the homogeneous polysaccharide isolated from *A. giraldii* Harms, known as AHP-II, and its immunomodulatory effects *in vivo* will be studied. High-performance ion chromatography (HPIC) and high-performance gel permeation chromatography (HPGPC) based analyses revealed that AHP-II was composed of various monosaccharides, which included rhamnose, arabinose, galactose, glucose, mannose, galacturonic acid, and glucuronic acid in molar ratios of 29.5 : 24.6 : 23.8 : 4.4 : 5.7 : 8.8 : 3.1, respectively, and had a collective molecular weight of 80.21 × 10^3^ Da. Fourier-transform infrared (FTIR) spectroscopy indicated the presence of a pyranose ring and *β*-type glycosidic linkages in AHP-II. In addition, immunomodulatory effect analyses of AHP-II that used a cyclophosphamide-induced immunosuppressive mouse model demonstrated that its treatment could significantly restore spleen and thymus indices, promote the proliferation of splenic lymphocytes, elevate CD4^+^ T lymphocyte percentage and CD4^+^ : CD8^+^ ratio in the spleen, promote macrophage phagocytosis, and restore cytokines (IL-6, TNF-*α*, IgM, and IgG) levels. These results suggested that AHP-II could potentially be used as natural immunomodulator and as an alternative treatment to reduce chemotherapy-induced immunosuppression.

## 1. Introduction

*Acanthopanax giraldii* Harms is a traditional Chinese medicine that is used by a number of ethnic minorities. In particular, its stem bark has been used to treat arthralgia due to wind, cold, and dampness, as well as weakness of the feet and knees [1], debilitating conditions in patients that have tumors or other chronic diseases [2]. Further analyses of *A. giraldii* stem bark revealed the presence of several active ingredients that include saponins, volatile oils, and a variety of polysaccharides. The main active ingredient is found in the polysaccharides. In addition, scientific studies indicated the antiviral, antitumor, and antiradiation effects of *A. giraldii* polysaccharides [3].

The immune system is the first line of defense against pathological diseases. It recognizes antigenic foreign bodies and subsequently removes them. It has an important role to maintain the stability of the internal environment and physiological balance in coordination with other systems [4, 5]. Immunosuppression, which is a temporary or permanent state of immune dysfunction, means that the body is more sensitive to pathogens [6]. Recent studies have indicated that certain polysaccharides found in Chinese herbal medicine have immune enhancing potential and could activate the immune system; therefore, the activity of immune cells that could subsequently produce antibodies against pathogens is promoted [7].

It has been shown that three types of polysaccharides (AHP-I, AHP-II, and AHP-III), extracted from *A. giraldii*, have an *in vitr*o activation effect on mouse peritoneal macrophages. Between these, AHP-II is the most effective [8]. In addition, studies have demonstrated that AHP-II activates mouse macrophages by binding to the TLR4 receptor and impacts the ERK, JNK/nuclear factor (NF-*κ*B) signaling pathways [9]. This study aims to investigate the structural characteristics of AHP-II and to systematically understand its immunomodulatory effect using a cyclophosphamide- (Cy-) induced immunosuppressive mice model. In addition, this study could potentially provide a theoretical basis for the development of *A. giraldii* as a type of immunopotentiator.

## 2. Materials and Methods

### 2.1. Materials

Lipopolysaccharide (LPS) and concanavalin A (Con A) were purchased from Sigma Aldrich (Saint Louis, Missouri, US), and cyclophosphamide (Cy) was obtained from Hengrui Medicine, Jiangsu province, China. *A. giraldii* was procured from Beijing Tong-Ren-Tang Group. Voucher specimens (WFMC602) were deposited at the School of Basic Medicine, Weifang Medical University, Weifang, China. ICR male BALB/c mice (8 weeks and 18–20 g) were purchased from the Animal Center of Shandong University, Jinan, China. The enzyme-linked immunosorbent assay (ELISA) kits were purchased from Bioscience (San Diego, CA, US), and the CCK-8 kit was purchased from Dojindo Molecular Technologies, Kumamoto, Japan.

### 2.2. Isolation and Purification of AHP-II

The extraction of *A. giraldii* polysaccharide (AHP) was performed as described in the literature [10]. Briefly, the polysaccharide was extracted from the dried bark of *A. giraldii*, which was identified by Professor Jinbao Tang (College of Pharmacy, Weifang Medical University, China); after mixing with distilled water in 8 : 1 (v/w) ratio, it was incubated at 58 °C for 70 min using 85 W ultrasonic power. This process was repeated three times. The extract was concentrated using a rotary evaporator, and the extraction solution was deproteinized using the Sevage method. Finally, the extracted solution was dialyzed for 72 h against distilled water (MWCO 7000 Da), and then 4 volumes of ethanol were added to precipitate the extracted AHP.

Then, AHP was solubilized in Tris-HCl buffer (0.05 mol/L pH 7.1) and purified with DEAE-52 cellulose (2.6 × 30 cm) column using gradient elution with approximately 0–0.8 mol/L NaCl Tris-HCl buffer (0.05 mol/L pH 7.1). The elution curves (AHP-a and AHP-b) are shown in Supplementary Figure S1. AHP-II was finally recovered from NaCl-eluted main fraction of AHP-b polysaccharide by further separation on Sephacryl S-300 HR (1.6 × 50 cm) column and elution with 0.1 mol/L NaCl, as shown in Supplementary Figure S2.

### 2.3. Molecular Weight Determination

The molecular weight of AHP-II was determined as described in the literature [11] using high-performance gel permeation chromatography (HPGPC (Agilent 1100 Series, Agilent, US)) that was equipped with a refractive index detector (2414 HPLC, Agilent, US) at 35 ^o^C. A SUGAR KS-802 column (8.0 mm × 30 cm, Shodex, Japan) was connected in series to another SUGAR KS-803 column (8.0 mm × 30 cm, Shodex) at 40°C. The 0.2 mol/L NaCl at 0.8 mL/min mobile phase was used. The sample solution (20 *μ*L) was injected into the machine after filtering with 0.22 *μ*m syringe filters. The T-series dextrans (T-10, T-40, T-70, T-110, T-500, and T-2000) molecular weight standards were used to generate the calibration curve.

### 2.4. AHP-II Monosaccharide Composition Analysis

The monosaccharide composition of AHP-II was detected and quantified using high-performance ion chromatography (HPIC). First, AHP-II was hydrolyzed with 2 mol/L trifluoroacetic acid (TFA) at 120°C in an ampoule bottle for 3 h. Then, the TFA was removed by nitrogen blow-drying, and the AHP-II sample was dissolved in deionized water. The chromatographic separation of monosaccharides was performed using ion chromatograph (IC (Thermo Fisher Scientific, ICS5000 type, MA, US)) that was equipped with a Dionex Carbopac™ PA20 column (150 mm × 3 mm) and pulse ampere detector. Further separation was performed by gradient elution at a flow rate of 0.3 mL/min. The mobile phase included the following reagents: A: water;B:15 mmol/L NaOH; and C:15 mmol/L NaOH and 100 mmol/L NaOAC. Finally, elution was carried out as follows: 98.8% A and 1.2% B at 0–18 min; 50% A and 50% B at 20–30 min; 100% C at 30.1–46 min; 100% B at 46.1–50 min; and 98.8% A and 1.2% B at 50.1–60 min.

### 2.5. Fourier-Transform Infrared Analysis of AHP-II

For this analysis, AHP-II was grounded with KBr powder and pressed into a 1 mm pellet for Fourier-transform infrared measurement using Fourier-transform infrared spectroscopy (FTIR) with a Shimadzu IR spectrometer (FTIR 8400S; Shimadzu, Tokyo, Japan).

### 2.6. Animals Studies

ICR male BALB/c mice were housed in a rodent facility with a 12 h dark/12 h light photoperiod and free access to water and a standard laboratory pellet diet. After 1 week of environmental adaptation, the mice were randomly classified into five groups (10 mice/group): (1) normal group (NG); (2) model group (MG); (3) AHP-II high-dose group (AHP-II-H); (4) medium-dose group (AHP-II-M); and (5) low-dose group (AHP-II-L). Mice in each group were intraperitoneally injected with 80 mg/kg dCy (0.2 mL), from days 1 and 3, except for mice in the NG group that received normal saline. The mice in the three AHP-II groups were given AHP-II at doses of 50, 100, and 200 mg/kg BW (body weight)/d (0.2 mL) by intragastric administration, respectively, from day 1 to day 10, and the NG and MG mice were treated with normal saline.

24 h after the final drug administration, the mice in each group were weighed and sacrificed and the spleens and thymuses were removed under aseptic conditions. After weighing the organs, the thymus and spleen indices (mg/g) were calculated as follows: weight of thymus or spleen (mg)/body weight (g).

### 2.7. Histochemical Examinations of the Spleen and Thymus

The spleens and thymuses that were extracted from the mice in each group were fixed overnight in 4% paraformaldehyde. Then, the tissue samples were embedded in paraffin blocks following dehydration and 4 *μ*m tissue sections were prepared. The slides were subsequently stained using hematoxylin and eosin [12] dyes, and the histological changes were examined using a light microscope (Olympus, Tokyo, Japan).

### 2.8. Peritoneal Macrophage Activity Assay

The peritoneal macrophages activity was assessed using chicken red blood cells (CRBCs) as described in the literature [13]. In summary, 30 min before sacrifice, the mice from each group were intraperitoneally injected with 1.0 mL of CRBCs suspension (5%, v/v, in PBS). After sacrifice, 2.0 mL of D-Hanks solution was injected for peritoneal lavage and then peritoneal exudates (0.2 mL) were smeared onto slides, which were at 37°C in a 5% CO_2_ humidified incubator for 2 h. Then, nonphagocytosed CRBCs and nonadherent cells were removed by washing in PBS and the macrophages were fixed with methanol for 5 min and stained with Giemsa dye for 3 min. The phagocytosis percentages (phagocytosis index) were determined by counting the number of phagocytosed CRBCs/100 macrophage cells.

### 2.9. Splenocyte Proliferation Assay

The spleens that were isolated from each group of mice were gently homogenized with PBS and passed through a 200-mesh filter to obtain a homogeneous splenocyte suspension. The erythrocytes were removed from the suspension using erythrocyte lysis buffer, and the remaining splenocytes were then suspended in complete RPMI-1640 media that contained 10% FBS, 100 *μ*/mL penicillin, and 100 *μ*g/mL streptomycin. Then, 2.0 × 10^5^ purified splenocytes were seeded into each well of a 96-well microplate and mixed with Con A (5 *μ*g/mL) or LPS (10 *μ*g/mL) in 200 *μ*L volume. The plates were then incubated at 37^o^C in a 5% CO_2_ humidified incubator for 48 h. Finally, the cellular proliferation was determined using a CCK-8 kit, as described in the literature [14].

### 2.10. Detection of CD4^+^ and CD8^+^ T Lymphocytes in Splenocytes

The splenocytes were incubated with labeled monoclonal antibodies (mAb) against CD3-FITC, CD8-APC, and CD4-PE (all antibodies were purchased from Lianke Biotech, Hangzhou, China) on ice for 60 min in the dark. Before analyses, the splenocytes were rinsed with PBS twice, and the ratio of CD4^+^ : CD8+ lymphocytes was analyzed by flow cytometry using the Guava EasyCyte Mini system (Guava Technologies, Chicago, Illinois, US).

### 2.11. Serum Cytokine Estimation

To estimate cytokine levels, whole blood was collected from eyeballs extracted from the mice. After 30 min, the blood samples were centrifuged at 4 °C at 3500 r/min for 10 min to obtain the serum. The concentrations of cytokines TNF-*α*, IL-6, IgG, and IgM from the serum were determined using ELISA assays (Bioscience, San Diego, CA, US).

### 2.12. Statistical Analysis

The quantitative data from all experiments are presented as mean ± SD. The statistical significance between different groups was analyzed using ANOVA with SPSS software. A *p* value of <0.05 represented a statistically significant difference.

## 3. Results

### 3.1. Characterization of AHP-II

**Figure 1:**
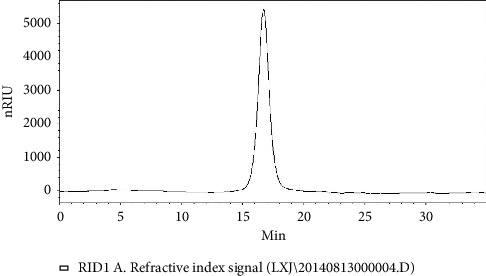
HPGPC analysis of AHP-II. The single peak corresponds to the purity of AHP-II.

**Figure 2:**
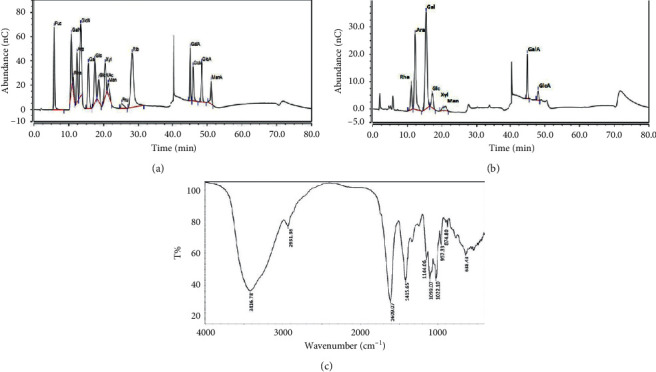
Structural characterization of AHP-II: (a) ion chromatogram of standard monosaccharide; (b) ion chromatogram of AHP-II; and (c) FTIR spectra of AHP-II.

HPGPC based analysis of AHP-II purity revealed a single peak shown in Figure 1. The molecular weight was approximately 80.21 kilodalton (kD). The monosaccharide compositions analysis of AHP-II by HPIC showed the presence of seven monosaccharides, rhamnose, arabinose, galactose, galactose, mannose, galacturonic acid, and glucuronic acid, based on the same retention time with monosaccharide standards on the chromatographic curve (Figures 2(a) and 2(b)). The molar ratios of rhamnose, arabinose, galactose, glucose, mannose, galacturonic acid, and glucuronic acid were identified as 29.5 : 24.6 : 23.8 : 4.4 : 5.7 : 8.8 : 3.1, respectively.

Then, AHP-II demonstrated no absorption at 260 and 280 nm in the UV spectrum, which confirmed the absence of proteins and nucleic acids. In addition, FTIR spectrum analysis of AHP-II revealed polysaccharide characteristic absorption peak spectra, as shown in Figure 2(c). In particular, a large intense peak was seen at approximately 3,416 cm^−1^, which represented the hydroxyl group, and a weak peak identified at approximately 2,931 cm^−1^ corresponded to absorption of C-H stretching vibration. In addition, the peak at approximately 1,609 cm^−1^ indicated the asymmetric stretching vibration of COO^−^, and the peak at 1,415.65 cm^−1^ indicated symmetrical COO^−^ stretching vibration. This indicated the presence of uronic acid [15]. In addition, the peaks at approximately 1,313 and 1,249 cm^−1^ appeared to have the characteristic absorption of C–H bonds. The peaks at 1,144, 1,099, and 1,022 cm^−1^ were due to asymmetric vibration of C-O-C glycosidic rings, which suggested that the skeletal modes of AHP-II included pyranose rings [16]. In addition, the absorption peak at 957 cm^−1^ depicted the characteristic absorption of pyranose, and the absorption peak at 874 cm^−1^ indicated the existence of *β*-configurations in the polysaccharides [17]. The results suggested that AHP-II had characteristic absorption properties similar to typical polysaccharides.

### 3.2. Impact of AHP-II on Spleen and Thymus Indexes

The spleen and thymus are important organs in the immune system of mice; therefore, their indices reflect the immune status to some degree [18]. In this study, compared with mice from the NG, the thymus and the spleen indices were significantly less in the MG mice (*p* < 0.01). However, spleen indices in AHP-II group were significantly increased (*p* < 0.01) compared with MG mice. Similarly, AHP-II (100 or 200 mg/kg) treatment showed significantly elevated (*p* < 0.01 or *p* < 0.05) thymus indices (Table 1). In addition, the mice treated with a low dose of AHP-II (50 mg/kg) displayed an improved thymus index; however, the difference was not significant compared with mice from the MG.

### 3.3. Effect of AHP-II on Macrophage Activation

The effect of AHP-II on macrophage activation was detected by performing peritoneal macrophage activity assays using CRBCs. The results showed that the phagocytosis percentage and phagocytosis index of the peritoneal macrophages from the MG mice were much lower than those of the NG mice (*p* < 0.01) (Table 2). However, mice in the AHP-II (50–200 mg/kg) treatment group displayed significantly improved phagocytic activities compared with mice from the MG group (*p* < 0.05 or *p* < 0.01) (Table 2). This data implied that AHP-II could activate macrophage function.

### 3.4. Effect of AHP-II on Mitogen-Induced Splenocyte Proliferation

**Figure 3:**
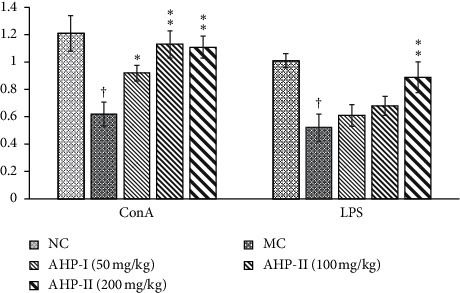
Effect of AHP-II on proliferation of mitogen-stimulated mice splenocytes. Proliferation analyses of splenocytes isolated from mice treated with ±AHP-II (50, 100, and 200 g/mL) and stimulated in vitro with 5 *μ*g/mL Con A or 10 *μ*g/mL LPS for 48 h using CCK-8 assay. Data represents the mean ± SD (*n* = 10). ^†^, *p* < 0.01 compared with NC group; ^*∗*^and ^*∗∗*^, *p* < 0.05 or *p* < 0.01 compared with MC group.

To understand the effect of AHP-II on splenocyte proliferation, isolated lymphocytes were treated with LPS and Con A, respectively, to induce differentiation into T and B lymphocytes. The proliferation of T and B lymphocytes that were isolated from the MG mice was significantly lower than that of those from the NG mice (*p* < 0.01), as shown in Figure 3. However, the proliferation of B lymphocytes isolated from the AHP-II treatment groups was significantly (*p* < 0.01or *p* < 0.05) higher compared with the MG mice and T cell proliferation was only significantly higher in mice treated with AHP-II (200 mg/kg dose). The 50 and 100 mg/kg treatment of AHP-II showed an increasing trend; however, the differences were not significant. In conclusion, the results showed that AHP-II had a synergistic effect with Con A or LPS treatment on splenocyte proliferation in the Cy-induced immunosuppressive mice.

### 3.5. Effect of AHP-II on Splenic T Lymphocyte Subsets

To understand the effect of AHP-II on cellular immunity, the numbers of CD4^+^ and CD8+ T splenic lymphocytes that were isolated from the different groups were analyzed by flow cytometry. As given in Table 3, the percentage of CD3+CD4+ T lymphocyte and the ratio of CD4+ : CD8+ were lower in the MG mice than in the NG mice (*p* < 0.01). In contrast, the percentage of CD3+CD4+ lymphocyte was significantly higher in mice in the AHP-II treatment groups (50, 100 mg/kg/d) compared with the MG mice (*p* *<* 0.01). In particular, the mice treated with AHP-II at 100 mg/kg dose displayed approximately the same percentage of CD3+CD4+ lymphocytes as the NG mice. In addition, a similar significant increase was observed in the ratio of CD4+ : CD8+ in the AHP-II treated mice. The results suggested that AHP-II treatment had immunomodulatory effects mainly through the activation of CD4+ T cells in Cy-induced immunosuppressive mice.

### 3.6. Effect of AHP-II on Levels of IgM, IgG, and Cytokines in Serum

To understand the immune-protective function of AHP-II on the humoral immune response, the levels of serum IgG and IgM were evaluated by ELISA. The results indicated that levels of IgG and IgM in the serum of the MG mice were much lower than those in the serum of the NG mice (*p* < 0.01) (Table 4). However, the serum of AHP-II treated immunosuppressive mice had significantly higher levels of IgM and IgG compared with the MG mice (*p* < 0.05 and *p* < 0.01, respectively) (Table 4). The TNF-*α* and IL-6 levels in the serum of the MG mice were lower than those in the serum of the NG mice (*p* < 0.01). However, the serum of AHP-II treated mice showed significantly upregulated levels of IL-6 and TNF-*α* in comparison with the MG mice (*p* < 0.01) (Table 4).

### 3.7. Impact of AHP-II Treatment on the Pathology of Thymus and Spleen

**Figure 4:**
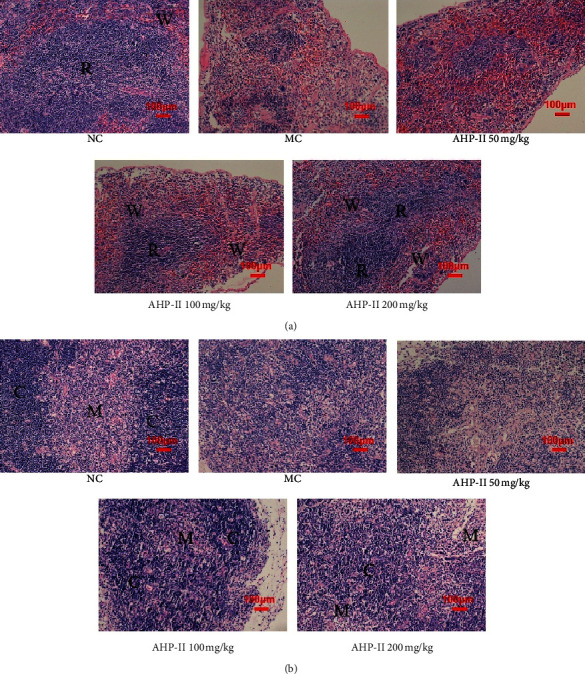
Effects of AHP-II on the morphology of spleen and thymus in Cy-induced immunocompromised mice using HE staining. HE staining pictures display morphology of (a) spleen and (b) thymus from represented mouse tissue sections obtained from Cy-induced immunocompromised mice treated with different concentrations of AHP-II (50, 100, and 200 mg/mL). R = red pulp area; W = white pulp area; C = cortex region; and M = medulla region. Scale bar = 100 *μ*m. NC: normal control; MC: model control, AHP-II: *A. giraldii* polysaccharide II.

The effects of AHP-II treatment on the pathology of the thymus and spleen were examined using hematoxylin and eosin (HE) staining of the tissue sections from the four treatment groups. As shown in Figure 4(a), HE staining of the spleens from the NG mice showed clear structures, a boundary between white pulp and red pulp, and closely arranged lymphocytes in the white pulp. In contrast, HE staining of spleen tissue sections from the MG mice demonstrated a smaller area of white pulp and a few lymphocytes. However, the medium-dose and high-dose AHP-II treatment significantly increased the white pulp area and the number of lymphocytes in the spleen sections, which had clear borders. The HE staining pattern of the spleen tissue sections from low-dose AHP-II-treated mice showed an increased number of lymphocytes; however, the difference was not significant compared with the patterns observed in sections from the MG group.

The HE staining of the thymus sections from the NG mice showed clearly demarked cortex and medulla, as shown in Figure 4(b). The cells in the cortex were dense with deep staining, and cells in the medulla were relatively sparse with light staining. The thymic corpuscles were scattered throughout the medulla. However, thymus sections from the MG mice showed a disordered thymus structure with no clear demarcation between the cortex and medulla. The thymus sections from mice treated with AHP-II showed some improvement in the thymus lesions, in particular, in the sections from mice treated with a medium-dose of AHP-II. In this group, the boundary between the cortex and medulla was significantly changed, and the number of lymphocytes in the cortex had increased. These findings suggest that medium and high doses of AHP-II could effectively improve spleen and thymus injury that was caused by cyclophosphamide, and a low dose of AHP-II had little impact.

## 4. Discussion

In this study, the characterization of the structural details of AHP-II and analysis of its immune regulatory effect using a Cy-induced immunosuppressive mice model were attempted. The structural analyses that used HPGPC, UV, HPIC, and FTIR techniques revealed that AHP-II was a polysaccharide with a molecular weight of 80.21 kD and had no other protein or nucleic acids components. It was composed of seven monosaccharides (rhamnose, arabinose, galactose, glucose, mannose, galacturonic acid, and glucuronic acid in the ratio of 29.5 : 24.6 : 23.8 : 4.4 : 5.7 : 8.8 : 3.1, respectively). This composition was different from previous experimental results [10] and could be attributed to improved detection methods.

The immune system is the first line of defense in any pathological disease, and immunosuppression (which refers to the vulnerability of the body to infection by bacteria, fungi, and viruses [6]) could have an adverse impact on overall body response to these infections [19]. Therefore, the immunomodulatory effect of AHP-II using Cy-induced immunosuppressive model was determined. Among all the body organs, the thymus and spleen are the major organs involved in the regulation of the immune responses. In particular, immune cells reside in the spleen and receive antigen stimulation to produce the immune response. Similarly, T lymphocyte differentiation and maturation occur along with peripheral T cell maturation in the thymus. Therefore, degeneration and atrophy of these organs could have a negative impact on the function of the entire immune system. The indices of the spleen or thymus could reflect the development of immune organs and the function of immune cells [20]. Therefore, this study showed that AHP-II treatment could improve the thymus and spleen indices in immunosuppressive mice and could positively improve pathological features.

The macrophages are among the various immune cells that play an important role in the defense against a pathological disease. Their activation improves the body's scavenging ability to clear away cancer cells and prevent infection [21]. The macrophages scavenge cells by phagocytosis. A previous study [22] showed that astragalus polysaccharide significantly increased phagocytosis in mouse peritoneal macrophages. In a previous study, it was shown that AHP-II could significantly increase the phagocytotic capacity of mouse peritoneal macrophages in vitro [8]. The results from this study show that AHP-II could significantly increase the phagocytosis percentage and index of peritoneal macrophages in Cy-induced immunosuppressive mice, which suggests that AHP-II could promote nonspecific immunity in an immunosuppressive environment.

In addition, lymphocyte proliferation is the most direct indicator of the immune status of the body. T and B lymphocytes participate in many important cellular and humoral immune responses. They are activated by stimulation from antigens or mitogens. Con A and LPS could upregulate T and B lymphocyte proliferation, respectively [23]. In this study, AHP-II treatment favored lymphocyte activation and proliferation, indicating that it could significantly ameliorate the lymphocytes responsiveness in Cy-induced immunocompromised mice and enhance the cellular immune system. In tumor cells, T lymphocytes mediate the antitumor immune response, and their activity is directly correlated with the occurrence and development of malignancies [24]. The CD4^+^ and CD8^+^ subsets are major T lymphocytes that participate in cellular immunity. Appropriate ratios and counts of CD4^+^ and CD8^+^ T lymphocyte subpopulations are important in immunoregulation. An altered equilibrium of T lymphocyte subgroups could lead to immune dysfunction, which could result in a series of immunopathological changes that affect the body's immune protection mechanisms [25]. The results from this study show that decreased CD4^+^ T cells and CD4^+^ : CD8^+^ ratio in Cy-treated model mice were consistent with the results in the literature [26, 27]. Of interest, AHP-II treatments reversed the Cy-induced decrease in the percentage of CD4^+^ and the ratio of CD4^+^ : CD8^+^ cells. This data indicated that oral administration of AHP-II could restore the immune function of immunosuppressed mice.

In addition, the impact of AHP-II treatment on IgG and IgM was analyzed, which normally participate in toxin neutralization and complement activation and opsonization [25]. Recent research indicates that various polysaccharides could enhance the humoral immune response through the promotion of specific IgM and IgG production [26]. In addition, AHP-II treatment significantly increased the levels of IgG and IgM in immunosuppressed mice, which suggested that it could potentially enhance humoral immunity.

Finally, the impact of AHP-II on cytokines secretion was analyzed, because they participate in and regulate various physiological and pathological processes, which include the immune response, cell differentiation, hematopoiesis, and tumor immunity and they are widely applied in the diagnosis, treatment, and prevention of diseases. Quantifying cytokine content could help to elucidate the immune regulatory function of AHP-II at the molecular level. In particular, IL-6 secretion was analyzed, which is responsible for enhancing the immune response of the innate immune system, promoting the removal of pathogenic bacteria, and stimulating the activated B cells to produce more immunoglobulin [27]. In addition, the TNF-*α* secretion was quantified, which promotes B cell differentiation, activates NK cells, and helps in the selective killing of tumor cells [28]. The analyses showed that AHP-II treatment significantly increased serum IL-6 and TNF-*α* levels in a dose-dependent manner in the Cy-induced immunosuppressive mice.

In conclusion, this study demonstrated that AHP-II polysaccharide is composed of rhamnose, arabinose, galactose, glucose, mannose, galacturonic acid, and glucuronic acid in the ratio of 29.5 : 24.6 : 23.8 : 4.4 : 5.7 : 8.8 : 3.1, respectively, with a pyranose ring and *β*-type glycosidic linkages. In addition, AHP-II exhibited potent immunomodulatory properties that could significantly improve immune organs (spleen and thymus) indices, macrophage phagocytosis, splenocyte proliferation, and levels of serum cytokines in immunosuppressive mice. These results implied that AHP-II could potentially improve the immune function in immunosuppressive mice. Therefore, AHP-II could potentially act as potent immunomodulatory drug that could be used in Cy-induced immunosuppression and in conjunction with other anticancer agents to protect and improve the immune function during chemotherapy.

## Figures and Tables

**Table 1:** Effect of AHP-II on spleen and thymus indices in Cy-induced immunosuppressive mice (*n* = 10, $\bar{x}\pm s$).

Group	Dose (mg/kg)	Spleen index (mg/g)	Thymus index (mg/g)
NC	0	3.72 ± 0.01	1.59 ± 0.15
MC	80	1.48 ± 0.08^*∗∗*^	0.47 ± 0.07^*∗∗*^
AHP-II	50	2.14 ± 0.11^††^	0.55 ± 0.05
	100	2.21 ± 0.50^††^	1.37 ± 0.28^†^
	200	2.11 ± 0.08^††^	1.41 ± 0.13^††^

NC = normal control; MC = model control; AHP-II = *A. giraldii* polysaccharide II. ^*∗*^ and ^*∗∗*^, *p* < 0.05 and *p* < 0.01 compared with NG; ^†^ and ^††^, *p* < 0.05 and *p* < 0.01 compared with MG.

**Table 2:** Effect of AHP-II on phagocytosis of peritoneal macrophage in Cy-induced immunosuppressive mice (*n* = 10, $\bar{x}\pm s$).

Group	Dose (mg/kg)	Phagocytic percentage (%)	Phagocytic index
NC	0	45 ± 3.6	0.83 ± 0.071
MC	80	26 ± 4.4^*∗*^	0.44 ± 0.099^*∗*^
AHP-II	50	35 ± 0.71^†^	0.84 ± 0.13^††^
	100	39 ± 1.1^†^	0.93 ± 0.183^††^
	200	45 ± 1.2^††^	1.09 ± 0.218^††^

NC = normal control; MC = model control; AHP-II = *A. giraldii* polysaccharide II. ^*∗*^, *p* < 0.01 compared with NG; ^†^ and ^††^, *p* < 0.05 and *p* < 0.01 compared with MG.

**Table 3:** Effect of AHP-II on splenic T lymphocyte subsets in Cy-induced immunosuppressive mice (mean ± SEM, *n* = 10).

Group	Dose (mg/kg)	CD4^+^ (%)	CD8^+^ (%)	CD4^+^ : CD8^+^
NC	0	56.65 ± 1.14	25.5 ± 1.54	2.22 ± 0.22
MC	80	49.55 ± 1.67^*∗∗*^	30.35 ± 0.88^*∗*^	1.65 ± 0.16^*∗∗*^
AHP-II	50	53.10 ± 2.13^††^	26.9 ± 1.97	1.97 ± 0.20^††^
	100	56.85 ± 2.62^††^	26.00 ± 1.22^†^	2.18 ± 0.18^††^
	200	46.1 ± 1.92	23.5 ± 1.77^††^	1.89 ± 0.14^††^

NC = normal control; MC = model control; AHP-II = *A. giraldii* polysaccharide II. ^*∗*^ and ^*∗∗*^, *p* < 0.05 and *p* < 0.01 compared with NG; ^†^ and ^††^, *p* < 0.05 and *p* < 0.01 compared with MG.

**Table 4:** Levels of serum IgM, IgG, and cytokines in Cy-induced immunocompromised mice (mean ± SEM, *n* = 10).

Group	Dose (mg/kg)	IgG (*μ*g/mL)	IgM (*μ*g/mL)	IL-6 (pg/mL)	TNF-*α* (pg/mL)
NC	0	785.32 ± 85.12	602.65 ± 38.5	138.44 ± 5.31	47.12 ± 5.62
MC	80	421.54 ± 45.31^*∗*^	214.41 ± 18.7^*∗*^	38.56 ± 4.11^*∗*^	25.79 ± 3.59^*∗*^
AHP-II	50	589.46 ± 65.27^††^	321.93 ± 26.7^††^	158.73 ± 6.23^††^	52.17 ± 3.57^††^
	100	658.33 ± 49.74^††^	383.37 ± 71.3^††^	162.38 ± 4.67^††^	59.22 ± 5.44^††^
	200	529.72 ± 80.31^†^	261.25 ± 60.6^†^	169.19 ± 4.21^††^	74.45 ± 4.79^††^

NC = normal control; MC = model control; AHP-II = *A. giraldii* polysaccharide II. ^*∗*^, *p* < 0.01 compared with control group; ^†^ and ^††^, *p* < 0.05 and *p* < 0.01 compared with MG.

## Data Availability

The data used to support the findings of this study are available from the corresponding author upon request.

## Supplementary Materials

Supplementary Materials Figure S1: elution curve of AHP on DEAE-52 cellulose column; AHP was applied to a column equilibrated with Tris-HCl buffer (0.05 mol/L pH 7.1) and with a linear gradient of 0–0.8 mol/L NaCl Tris-HCl buffer (0.05 mol/L pH 7.1). The fractions (AHP-a and AHP-b) were assayed using the phenol–H_2_SO_4_ reaction to estimate sugar content (OD 490 nm). Figure S2: elution curve of AHP-b on Sephacryl S-300 HR column; AHP-b fraction was applied to the Sephacryl S-300 HR column equilibrated with 0.1 mol/L NaCl. The fractions (AHP-II and AHP-III) were assayed using the phenol-H_2_SO_4_ reaction to estimate sugar content (OD 490 nm).
