# Supplementary figures and images for: Analysis of Acanthopanax giraldii Harms Polysaccharide II Composition and Its Immune-Protective Role in a Cyclophosphamide-Induced Immunosuppressive Mice Model
Source: Evid Based Complement Alternat Med. 2021 Jul 31;2021:3387396. doi: 10.1155/2021/3387396 (PMC8349253; doi:10.1155/2021/3387396)

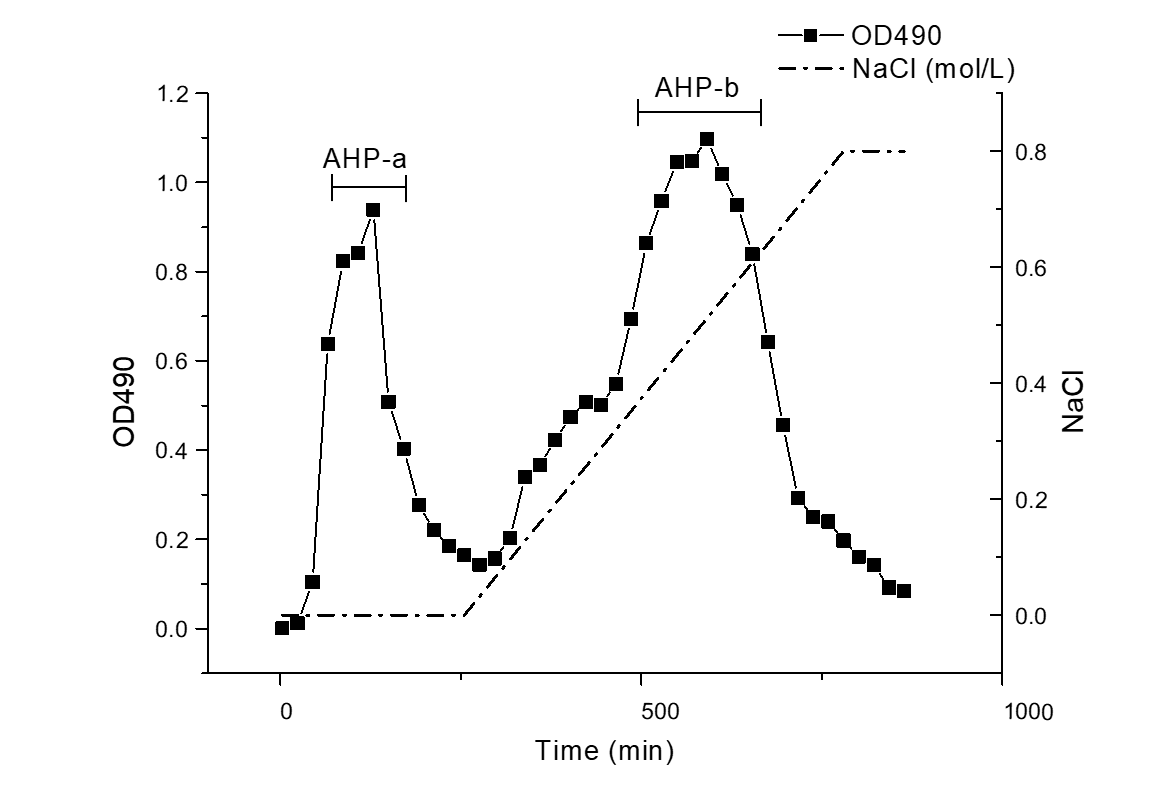

Supplement: Supplementary Materials — Figure S1: elution curve of AHP on DEAE-52 cellulose column; AHP was applied to a column equilibrated with Tris-HCl buffer (0.05 mol/L pH 7.1) and with a linear gradient of 0–0.8 mol/L NaCl Tris-HCl buffer (0.05 mol/L pH 7.1). The fractions (AHP-a and AHP-b) were assayed using the phenol–H2SO4 reaction to estimate sugar content (OD 490 nm). Figure S2: elution curve of AHP-b on Sephacryl S-300 HR column; AHP-b fraction was applied to the Sephacryl S-300 HR column equilibrated with 0.1 mol/L NaCl. The fractions (AHP-II and AHP-III) were assayed using the phenol-H2SO4 reaction to estimate sugar content (OD 490 nm). [file 3387396.f1.zip › 3387396.f1/Supplementary Figure 1.docx]

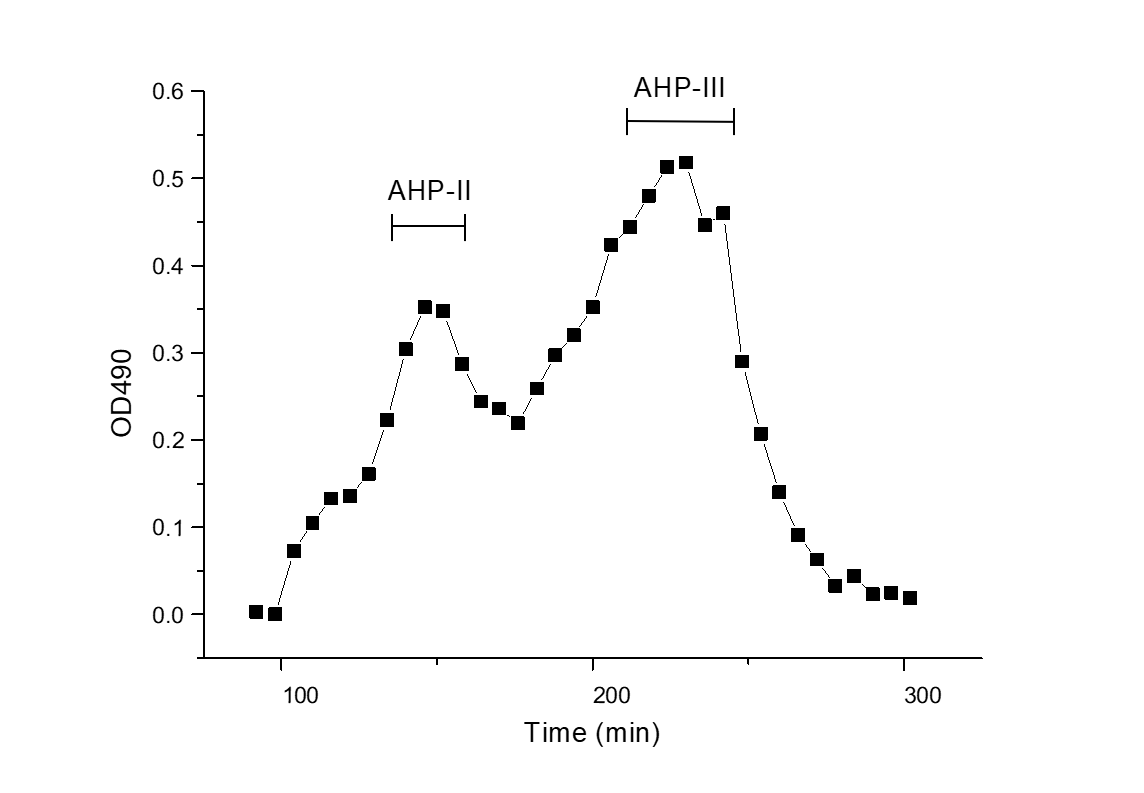

Supplement: Supplementary Materials — Figure S1: elution curve of AHP on DEAE-52 cellulose column; AHP was applied to a column equilibrated with Tris-HCl buffer (0.05 mol/L pH 7.1) and with a linear gradient of 0–0.8 mol/L NaCl Tris-HCl buffer (0.05 mol/L pH 7.1). The fractions (AHP-a and AHP-b) were assayed using the phenol–H2SO4 reaction to estimate sugar content (OD 490 nm). Figure S2: elution curve of AHP-b on Sephacryl S-300 HR column; AHP-b fraction was applied to the Sephacryl S-300 HR column equilibrated with 0.1 mol/L NaCl. The fractions (AHP-II and AHP-III) were assayed using the phenol-H2SO4 reaction to estimate sugar content (OD 490 nm). [file 3387396.f1.zip › 3387396.f1/Supplementary Figure 2.docx]
